# Adipose tissue macrophages: implications for obesity-associated cancer

**DOI:** 10.1186/s40779-022-00437-5

**Published:** 2023-01-03

**Authors:** Bei Li, Si Sun, Juan-Juan Li, Jing-Ping Yuan, Sheng-Rong Sun, Qi Wu

**Affiliations:** 1 Department of Pathology, Renmin Hospital of Wuhan University, Wuhan, 430060 China; 2 Department of Clinical Laboratory, Renmin Hospital of Wuhan University, Wuhan, 430060 China; 3 Department of Breast and Thyroid Surgery, Renmin Hospital of Wuhan University, Wuhan, 430060 China; 4 Tongji University Cancer Center, Shanghai Tenth People’s Hospital of Tongji University, School of Medicine, Tongji University, Shanghai, 200092 China

**Keywords:** Adipose tissue macrophages, Macrophage, Adipose, Obesity, Cancer, Therapy

## Abstract

Obesity is one of the most serious global health problems, with an incidence that increases yearly and coincides with the development of cancer. Adipose tissue macrophages (ATMs) are particularly important in this context and contribute to linking obesity-related inflammation and tumor progression. However, the functions of ATMs on the progression of obesity-associated cancer remain unclear. In this review, we describe the origins, phenotypes, and functions of ATMs. Subsequently, we summarize the potential mechanisms on the reprogramming of ATMs in the obesity-associated microenvironment, including the direct exchange of dysfunctional metabolites, inordinate cytokines and other signaling mediators, transfer of extracellular vesicle cargo, and variations in the gut microbiota and its metabolites. A better understanding of the properties and functions of ATMs under conditions of obesity will lead to the development of new therapeutic interventions for obesity-related cancer.

## Background

As an increasingly serious global epidemic, increasing evidence from clinical and preclinical studies suggests that obesity is a preventable risk factor for cancer morbidity and mortality, and results in approximately 20% of adult cancer-associated deaths [1–3]. In particular, obesity-induced chronic inflammation plays a promoting role in cancer initiation and progression via its ability to form a permissive microenvironment for neoplastic transformation [4]. For example, in the PC-3 M-Luc-C6 prostate cancer mouse model, high-fat diet-induced obesity activated the macrophage inhibitory cytokine-1 signaling pathway by metabolically increasing adipose lipolysis and free fatty acid release, which further elevated the expression of IL-8 and IL-6 and subsequently advanced prostate cancer progression [5]. Although some studies have focused on obesity, the impact of obesity on cancer initiation and progression remains unclear. Elucidating the core pathological characteristics of adipose tissue may provide crucial insights for understanding the crosstalk between obesity and cancer.

Adipose tissue exists extensively in various anatomical locations, and there are different adipose tissue depots, including subcutaneous and visceral white adipose tissue, brown adipose tissue, inter- and intra-muscular adipose tissue, marrow adipose tissue, and dermal adipose tissue [6]. Adipose tissue is thought to be composed of adipocytes and diverse nonadipocyte cells, including pericytes, endothelial cells, monocytes, macrophages, and adipose-derived stromal/stem cells [7, 8]. Adipose tissue macrophages (ATMs) are a hybrid of macrophages composed of tissue-resident macrophages and myeloid monocytes. Crown-like structures (CLSs) are a representative configuration of macrophages in adipose tissues, in which macrophages surround and phagocytose a dead or dying adipocyte [9]. Notably, the number and density of CLSs are positively correlated with a high body mass index, large adipocyte size, postmenopausal status, and insulin resistance in obese subjects. Compared with lean mice, CLS counts were 5.2-fold greater in obese mice, and CLSs were closer to the tissue center in obese mice [10]. In patients with breast cancer, the pathological elevation of CLSs in mammary adipose tissue was associated with inferior prognosis and aggressive behavior [11]. Therefore, understanding the role of ATMs is essential for obesity-associated cancer research.

Notably, the macrophage phenotype can vary among different cancer types and intratumor regions [12]. First, the reprogramming of macrophages from one phenotype into another partly explains the diversity of macrophages [13]. In response to different environmental cues, ATMs change their transcriptional programs to ‘polarize’ from a homeostatic state into an inflammatory state or vice versa [14, 15]. Subsequently, the crosstalk between macrophages and other cells, such as fibroblasts [16], endothelial cells [17], and adipocytes [18], contributes to the alteration of ATM phenotypes. For example, exosomes derived from adipose-derived stromal/stem cells promote macrophage polarization to an alternatively polarized phenotype, thereby facilitating angiogenesis [19]. In contrast, macrophages, especially classically polarized macrophages, secrete proinflammatory cytokines, such as vascular endothelial growth factor α, which activate endothelial cells and facilitate proliferation and blood vessel development [20, 21]. Therefore, understanding multifunctional macrophages and the intersection between macrophages and other cells is essential for obesity-associated cancer research.

Hence, we review these primary factors mediating the functionality of ATMs, including metabolism, inflammatory factors and extracellular vesicles. We also investigate the effects of these factors on obesity-related cancer progression mainly in tumor types with a high composition of adipose tissue (e.g., breast cancer).

## Origin, feature, and function of ATMs

Evidence has demonstrated that macrophages are derived from three different pathways, including embryonic or adult hematopoietic stem cell (HSC) progenitor cells, as well as monocytes [22]. Tissue-resident macrophages originate from embryonic precursors or adult HSCs, with the relative contributions of these populations varying by tissue. Likewise, monocyte-derived macrophages account for a certain proportion of several tissues under inflammatory conditions. Importantly, macrophages in adipose tissues have a mixed origin [23]. In general, most ATMs are thought to be derived from the embryonic origin (also named tissue-resident macrophages) in homeostatic conditions, when there is an extensive accumulation of monocytes that differentiate into macrophages later in obesity-related adipose tissues [24]. In solid tumors, the proportions of monocyte-derived macrophages and tissue-resident macrophages vary by tumor type. In cervical and mammary tumors, recent evidence has shown that tumor-infiltrating macrophages usually originate from monocytes [25]; however, other studies revealed that tissue-resident macrophages account for 50% of the macrophage populations in pancreatic ductal adenocarcinoma [26]. Moreover, the proportion also changes with tumor development. Tissue-resident macrophages dominate in the early stage of tumorigenesis; however, during tumor growth, tissue-resident macrophages were gradually replaced by monocyte-derived macrophages in the mouse and human non-small cell lung carcinoma microenvironment [27]. Hence, several novel approaches, such as single-cell RNA sequencing (scRNA-seq), should be applied to explore the origin and impact of ATMs in human cancers.

Abundant evidence has revealed significant heterogeneities among ATMs, and the features of different ATMs have been described by scRNA-seq [28–30]. In visceral adipose tissue, ATMs are further classified into two monocyte and three macrophage subtypes. The two monocyte subsets, Mon1 and Mon2, differ by the expression of *Retnla, Fn1* (Mon1), *Plac8*, and *Clec4e* (Mon2). One macrophage subtype (Mac1) was characterized by a high expression of *Retnla, Cd163, Lyve1*, and *Cd209f*, while the other macrophage subtype (Mac2) mainly overexpresses *Cd9* and *Nceh1* [28]. In addition, a new type (Mac3) of macrophages was identified, lipid-associated macrophages (LAMs). They are differentiated from monocytes and express *Trem2, Lipa, Lpl, Ctsb, Ctsl, Fabp4, Fabp5, Lgals1, Lgals3, Cd9* and *Cd36*, which are closely related to lipid metabolism and phagocytosis [28]. In subcutaneous fatty tissue, three distinct ATM subsets were identified, lymphocyte antigen 6C (Ly6C^+^) and two different Ly6C^–^ (CD9^+^Ly6C^–^ and CD9^−^Ly6C^–^) subgroups [31].

Generally, embryonic-derived macrophages in tumor tissues function in tissue remodeling and wound healing [26], while macrophages derived from HSCs exert an immunosuppressive effect [32]. Apart from this, the ATM subtype has very different functions. Among them, LAMs mainly accumulate in tumor-adipose junctional regions and lung metastatic lesions in mammary tumors, which contribute to tumor growth and metastasis [30, 33]. Interestingly, obesity led to a significant increase in lipid metabolism-related genes in ATMs expressing *Lpl, Trem2*, and *Cd9* but a significant decrease in macrophage-specific genes [34, 35]. Thus, LAMs may be a link between obesity and malignant behaviors. Additionally, Ly6C^+^ ATMs in subcutaneous fatty tissue are mainly distributed outside the CLS and enable it to accelerate adipogenesis, while CD9^+^ ATMs are located within the CLS, which are rich in lipids and proinflammatory. Thus, diverse ATMs exert multitudinous effects on tumor growth and development.

## Polarization of ATMs

Macrophages have great plasticity, as evidenced by diverse polarization patterns, which further form multiple functional phenotypes in response to various environmental cues. Two distinct polarization types for macrophages have been clarified: the classically polarized (M1) macrophage and the alternatively polarized (M2) macrophage [36]. Macrophages are activated toward the M1 phenotype in response to interferon-gamma (IFN-γ) or bacterial moieties such as lipopolysaccharide (LPS). In contrast, macrophages are polarized toward the M2 subtype upon stimulation with IL-4 [37]. The main features of M1 macrophages are the secretion of proinflammatory cytokines, increased cytotoxic activity against bacteria and viruses, and facilitation of antitumor immunity [38]. However, M2 macrophages have an anti-inflammatory effect and are detected in allergies, parasitic infections, tissue remodeling, and tumor development [39]. Additionally, M2 macrophages exhibit the generation of ornithine and polyamines via the arginase pathway, increased generation of scavenging, galactose, mannose receptors, and secretion of anti-inflammatory cytokines (including IL-4 and IL-10) as well as chemokines [chemokine (C-C motif) ligand (CCL)17, CCL22 and CCL24] [12]. M2 macrophages also promote angiogenesis and attract Th2 and T regulatory cells to inevitably have immunosuppressive functions [40].

Crucially, ATMs can be activated into a switching phenotype, which secretes both proinflammatory and anti-inflammatory cytokines and is also found in cancers, such as malignant mesothelioma [41]. These macrophages may be self-reprogramming or undergoing a polarization shift, leading to mutual immunosuppression by the alternative phenotype [42]. Macrophages in intermediate or overlapping states have been detected in vivo under pathophysiological conditions with diverse and temporally changing activating signals. For instance, CD11c^+^ ATMs derived from obese mice also show an overlapping profile, with the increased transcription of both M1- and M2-associated genes [14]. These observations demonstrate the different polarized phenotypes of macrophages and show that the typical M1 and M2 phenotypes are extremes of macrophage functional states [43].

## Regulatory mechanisms of macrophage polarization in adipose tissue and cancer progression

Apart from M1/M2 macrophage phenotypes, evidence demonstrates that there is a more complex scenario dictating macrophage functional states in adipose tissues [37]. Increasing evidence indicates that macrophage polarization has a vital effect on the progression of obesity and obesity-associated cancer [44, 45]. However, the main mechanisms that result in macrophage polarization in adipose tissues and obesity-related cancers are still ambiguous. Thus, the following sections discuss the integrated mechanisms of obesity-associated macrophage polarization in either adipose tissues or both adipose tissues and tumor physiology: metabolic dysregulation; secreted molecules, including chemokines, cytokines, adipokines, and other inflammatory factors; extracellular vesicles (EVs); and gut microbes.

### Metabolic dysregulation

**Fig. 1:**
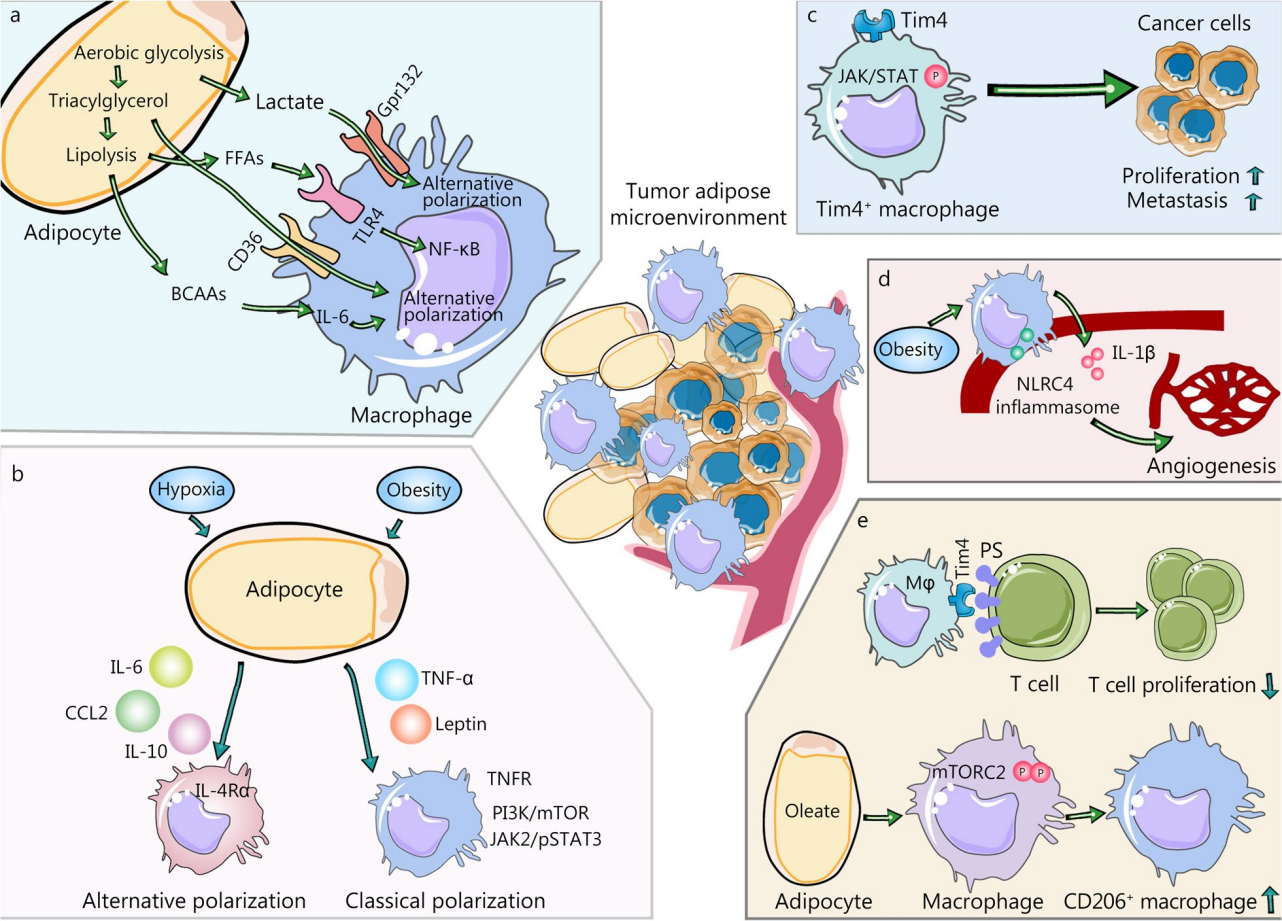
Polarization of ATMs is induced by metabolites and inflammatory factors derived from tumor adipose microenvironment (TAME). **a** Function and polarization of macrophages in TAME are regulated by metabolites, derived from glycolysis, lipid metabolism, and amino acid metabolism. Lactate from aerobic glycolysis promotes the alternative polarization of macrophages. Free fatty acids (FFAs) activate the toll-like receptor (TLR) 4 and increase the expression of proinflammatory genes dependent on nuclear factor kappa B (NF-κB). Branched-chain amino acids (BCAAs), another metabolite from triacylglycerol lipolysis, participate in inducing immune-suppressing macrophages. **b** Polarization of ATMs is also regulated by inflammatory factors. For example, leptin activated the JAK2/pSTAT3 pathway to function as a proinflammatory factor. Leptin-activated phosphatidylinositol 3-kinase (PI3K)/mammalian target of rapamycin (mTOR) pathway in macrophages to increase the production of lipid droplets and induce the classical polarization of macrophages. In contrast, M2 stimuli such as IL-6, IL-10, and CCL2, trigger alternative macrophage polarization. CCL2 promotes the production of IL-10 and increases the generation of M2-associated markers. **c** Tumor macroenvironment has a fundamental effect on tumor growth and metastasis. Tim4^+^ ATMs have a high level of activation of JAK/STAT signaling and promoted the progression and metastasis of ovarian cancer. **d** Obesity results in the activation of NLRC4 inflammasome in macrophages to increase their infiltration and the generation of IL-1β. In turn, IL-1β contributes to elevated angiogenesis. **e** Though integrating with CD8^+^ T cells with PS overexpression, Tim4^+^ macrophages suppress their proliferation and make them away from tumor targets. Oleate, one of the long-chain unsaturated fatty acids, amplifies the immunosuppressive effects of TAMs by inducing the polarization of macrophages into the CD206^+^ suppressive subtype by hyper-phosphorylating mTORC2. ATMs adipose tissue macrophages, BCAAs branched-chain amino acids, CCL2 chemokine (C-C motif) ligand 2, IL-1β interleukin-1beta, IL-10 interleukin-10, IL-6 interleukin-6, mTORC2 mTOR complex 2, NF-κB nuclear factor kappa B, NLRC4 NOD-like receptor (NLR) family CARD-containing protein 4, PS phosphatidylserine, TAM tumor-associated macrophage, TLR toll-like receptor, TNF-α Tumor necrosis factor-α, TNFR TNF-α receptor

Obesity-induced pathological changes have an important impact on cell metabolism. Macrophage polarization may be stimulated by various metabolic intermediates caused by obesity. With different stimuli, macrophages display different metabolic preferences that impact their differentiation, polarization, mobilization and the establishment of effective antitumor response capabilities. For example, LPS- and/or IFN-γ-stimulated M1 macrophages exhibit elevated glucose uptake and aerobic glycolysis, while IL-4-stimulated M2 macrophages are more prone to oxidative phosphorylation and fatty acid oxidation [46]. However, the metabolic preference for ATMs is not clear. Several major metabolites that stimulate macrophage activation and function in adipose tissues have been identified, as detailed below (Fig. 1a).

#### Glycolysis

Glycolysis is the decomposition of glucose into pyruvate under anaerobic or hypoxic conditions [47]. Importantly, hypoxia is a major characteristic of hypertrophic adipose tissue or the tumor microenvironment (TME). Hypoxia-driven glycolysis enables the recruitment of macrophages and activates their functions via diverse mechanisms. First, lactate is one of the main metabolites during glycolysis and has a crucial effect on ATMs. Malignant cells secrete high amounts of lactate into the extracellular environment due to the significant consumption of glucose [48]. Likewise, lactate derived from endothelial cells was taken up by macrophages in a monocarboxylate transporter 1-dependent manner [49]. Lactate in the microenvironment mainly contributes to the alternative polarization of macrophages and their functions [50]. Mechanistically, lactate binds to histone lysine residues to promote histone lactylation and then induces a switch to an M2-like phenotype in the late phase [51]. In addition, by directly binding to mitochondrial antiviral-signaling protein, lactate inhibited interferon-mediated pathways and alternatively promoted macrophage polarization indirectly [52]. Lactate can also activate G protein-coupled receptor 132 (Gpr132) on macrophages to facilitate macrophage alternative polarization, which further promotes the migration and invasion of breast cancer cells; accordingly, the deletion of Gpr132 reduces M2 macrophages and impairs breast cancer lung metastasis in mice [53]. Thus, high levels of lactate in hypertrophic adipose tissue stimulate protumorous functions and the alternative activation of macrophages. Furthermore, as a proinflammatory metabolite, succinate stabilizes hypoxia-inducible factor 1α (HIF-1α) by suppressing the activity of prolyl hydroxylase to increase glycolysis [54]. Metabolomic analysis of LPS-activated macrophages revealed an increase in aerobic glycolysis and a decrease in tricarboxylic acid cycle (TCA cycle) intermediates, which were directly related to succinate expression [54]. Therefore, succinate may be an important regulator in macrophage polarization by enhancing aerobic glycolysis.

#### Lipid metabolism

Adipose tissue is composed of large lipid droplets, which are a necessary form of energy storage and regulate lipid metabolism. Macrophages in hypertrophic adipose tissue facilitate lipid storage to form specific macrophage foam cells [55]. These macrophages have elevated inflammatory potential, which in turn exacerbates a positive inflammatory loop in adipose tissue [56]. Under obesity-induced lipid metabolic disorders, ATMs are capable of promoting lysosome biogenesis to accumulate lipid droplets [57]. However, the origin of lipids in ATMs remains unknown. Recent study has revealed that obese adipocytes enable the release of a large amount of lipid-filled exosomes, which provide lipids for macrophages [58]. Then, adipocyte-derived lipid-filled exosomes and relevant factors were sufficient to induce the differentiation of bone marrow precursors into adipose tissue macrophage-like cells in vitro[58]. Hence, the increased infiltration of lipid-overloaded macrophages was discovered in obese adipose tissues. Additionally, Tim4^+^ Lyve1^+^ ATMs exhibit a high level of lipid uptake and metabolism, with high lysosomal activity and cholesterol efflux [59]. Furthermore, lipid metabolites such as triacylglycerol and free fatty acids (FFAs) contribute to plastic changes in macrophages via the activation of stress and inflammatory pathways. First, triacylglycerol substrates are internalized by M2-polarized macrophages via scavenger receptor cluster of differentiation 36 (CD36), subsequently undergoing lipolysis by lysosomal acid lipases [60, 61]. Likewise, alternatively polarized macrophages can take up oxidated fatty acids and increase mitochondrial biogenesis [62]. Additionally, FFAs derived from entrapped adipocytes could activate the toll-like receptor (TLR) 4 on the plasma membrane of macrophages, upregulating the expression of proinflammatory genes dependent on nuclear factor kappa B (NF-κB) [63]. Finally, the dominant effectors involved in fatty acid oxidation are transcriptionally activated by peroxisome proliferator-activated receptor-gamma (PPARγ) and its downstream effector PPARγ-coactivator-1β [62, 64]. PPARγ plays an important role in alternative or protumorigenic polarization of macrophages both in vivo and in vitro [62]. Additionally, PPARγ-coactivator-1β is pivotal for macrophage polarization toward the M2 phenotype [64].

#### Amino acids

Adipose tissue has also emerged as a key regulator of amino acid metabolism [65]. Multiple amino acids derived from adipose tissues stimulate the polarization and function of macrophages. Originally, glutamate was found to be increased in plasma in obese individuals [66]. Glutamine replenishes intermediate catabolites for the TCA cycle and provides nitrogen and carbon for protein, amino acid, nucleotide, and lipid synthesis [67]. Glutamine stimulates the generation of inflammatory cytokines in macrophages [68]. The potential mechanism is glutamate-produced TCA cycle anaplerosis, which has a key impact on macrophage activation [69]. Integrated high-throughput transcriptional-metabolic profiling and analytical studies indicated that M2-polarized macrophages exhibit enhanced glutamine catabolism and activate the hexosamine biosynthesis pathway [69]. Correspondingly, both M2 polarization and the expression of M2-specific markers are inhibited by the deprivation of glutamine or inhibition of N-glycosylation. Nevertheless, glutamine is not required for the activation of classically polarized macrophages induced by LPS [69].

In addition, tryptophan is also one of the major amino acids that stimulate macrophage polarization. The increased production of tryptophan catabolites, including kynurenine, is associated with the pathophysiology of obesity [70]. It is worth noting that tryptophan catabolism in macrophages suppresses the activity of the adaptive immune system [47]. LPS, IFN-γ, and activation of TLRs regulate the expression of indoleamine 2,3-dioxygenase, a rate-limiting enzyme in tryptophan catabolism, and promote flux along the kynurenine pathway. Kynurenine, an agonist of the aryl hydrocarbon receptor, can be released by macrophages and strongly regulates the response of effector T cells and T regulatory cells to both inflammation and antigens [71].

Ultimately, large amounts of branched-chain amino acids (BCAAs) are metabolized by adipose tissue [65], and elevations in the concentrations of circulating BCAAs are significantly associated with obesity [72]. BCAAs, including leucine, valine, and isoleucine, also have an impact on macrophage activation. Specifically, BCAAs decrease the expression of IL-6 in macrophages and then attenuate proinflammatory function [73]. Branched-chain aminotransferase (BCAT) is the enzyme that initiates BCAA catabolism. As the main isoform of BCAT, BCAT1 is highly expressed in macrophages and stimulates oxygen consumption, glycolysis, immune responsive gene 1 (IRG1) levels, and the synthesis of itaconate [74]. Long-term treatment with ERG240, a leucine analog, inhibits the activity of BCAT1 and decreases the production of IL-1β in mice. This finding indicates that ERG240 could elevate BCAA levels and reduce IL-1β by suppressing the IRG1/itaconate axis [74]. Similarly, miRNA-93 reduces the production of IRG1-itaconic acid, thereby inhibiting the M2-like polarization of macrophages [75]. In db/db mice, long-term high BCAA supplementation further increases the levels of branched-chain alpha-keto acid (BCKA, a metabolite of BCAA) and results in macrophage hyperactivation [76]. Elevated BCKAs, not BCAAs, promote the generation of cytokines in primary macrophages [76]. BCKAs derived from glioblastoma can be absorbed and re-animated to BCAAs by tumor-associated macrophages (TAMs) [77]. Then, the phagocytic activity of macrophages decreases. This study indicates that tumor-derived BCKAs may play a direct role in the polarization of TAMs [77]. These studies demonstrate that BCAAs participate in the immunosuppressive function of macrophages, while BCKAs mainly play a role in the formation of the inflammatory microenvironment. Therefore, glutamine, tryptophan, and BCAAs alter macrophage functions in various ways.

### Abnormally secreted molecules

Due to the accumulation of metabolites in the cell and the formation of hypoxic areas, the secretion of cytokines and adipocytokines in adipose tissue emerges to become dysfunctional. Under such conditions, the diverse products derived from adipose tissue may also influence the function and polarization of macrophages. In this section, we summarize the alterations of these factors resulting from adipose tissue dysfunction and how they affect macrophage polarization (Fig. 1b).

#### Cytokines and chemokines

In obese conditions, the hypertrophic expansion of adipose tissue has many common features with the growth of solid tumors. Hypoxia in obese adipose tissue as well as tumorous tissues induces the expression of the transcription factor HIF-1α to stimulate the high expression of proinflammatory cytokines, including IL-6, tumor necrosis factor-α (TNF-α) and CCL2 [78, 79]. IL-6 is involved in various biological activities, including immune regulation, hematopoiesis, and tumorigenesis [80]. In the mammary adipose tissue of humans and mice with obesity, IL-6 can be secreted by macrophages with a proinflammatory phenotype in an NADPH oxidase 2-dependent manner. Furthermore, IL-6 stimulates macrophage polarization toward the M2 phenotype and excessive proliferation via the upregulation of IL-4 receptor α (IL-4Rα) [81]. IL-6 facilitates the stem-like properties of breast cancer cells by interacting with glycoprotein 130 [82]. Second, TNF-α released from tumor and stromal cells acts as a critical inflammatory factor in the obesity-induced microenvironment. A recent study showed that macrophages derived from bone marrow from IL-1β^−/−^ mice underwent M1 polarization, while TNF-α deletion inhibited M1 macrophage polarization [83]. In mice with knockout of TNF-α receptors (TNFR1 and TNFR2) (RKO), adipose tissue in high-fat/sucrose-diet-fed RKO mice exhibited elevated infiltration of macrophages; however, compared with those in wild-type mice, the macrophage phenotype markers in RKO mice were characterized by anti-inflammatory M2 over proinflammatory M1 markers [84]. Thus, TNF-α has a vital effect on the classically polarized phenotype of macrophages to promote inflammatory reactions. The CCL2/C-C chemokine receptor 2 (CCR2) pathway derived from hypertrophic adipocytes is enabled to recruit more macrophages in obese adipose tissue [61]. Subsequently, CCL2 upregulates the mannose receptor (CD206) in stimulating CD11b^+^ cells, and CD206 is a classical marker of M2-polarized macrophages [85]. Likewise, CCL2 promotes the generation of IL-10 induced by LPS in macrophages, while the blockade of CCL2 leads to an increased level of M1-associated genes and reduces the production of M2-associated markers in human macrophages [86]. Therefore, CCL2 not only recruits macrophages to the microenvironment but also shapes the M2-like polarization of macrophages. Moreover, IL-33 and IL-4 promote the alternative polarization of macrophages through metabolic reprogramming in a transcription factor GATA3-dependent manner [87]. Specifically, IL-4 induces de novo adipogenesis by activating the transcription of sterol regulatory element-binding protein 1. During the process of lipogenesis, a large amount of NADPH is consumed, and the levels of reactive oxygen species are elevated, which acts as a secondary messenger to signal adequate de novo lipogenesis and promote alternative polarization of macrophages [88].

#### Adipokines

Adipokine disorder emerges in obese individuals, commonly manifesting as upregulated secretion of leptin and resistin but decreased secretion of adiponectin. Leptin is now at the center of the association between obesity and cancer because it is generated in proportion to fat mass [89]. Leptin leads to the elevated secretion of IL-6 and IL-1β, demonstrating that leptin seems to facilitate the generation of proinflammatory factors by macrophages [90]. Likewise, leptin injection induced the activation of macrophages in TNF-α- and chemokine (C-X-C motif) ligand (CXCL) 1-dependent manners [91]. Mechanistically, leptin activates the JAK2/pSTAT3 pathway to upregulate the expression of chemokines to function as a proinflammatory factor [92]. Leptin also indirectly regulates macrophages through mast cell signaling. Recently, a study showed that leptin-deficient mast cells induce a swift from M1 to M2 for macrophages due to impaired cell signaling and changes in the balance between proinflammatory and anti-inflammatory cytokines [93]. From metabolic aspects, leptin-stimulated macrophages exhibit an increased production of lipid droplets through activation of the phosphatidylinositol 3-kinase (PI3K)/mammalian target of the rapamycin (mTOR) pathway [94]. Taken together, these findings demonstrate that leptin can trigger the macrophage M1 phenotype and proinflammatory cytokine production. In contrast, adiponectin is mainly produced by adipose tissue but has an anti-inflammatory impact by suppressing the generation of TNF-α and IL-6 [95]. Furthermore, adiponectin promotes the production of M2 markers but has a reverse effect on the expression of M1 markers in macrophages derived from human monocytes and stromal vascular fraction cells derived from human adipose tissue [96]. These observations demonstrate that adiponectin facilitates anti-inflammatory M2 polarization of macrophages.

Resistin is another predominant adipokine. Resistin has been shown to regulate glucose metabolism and exert proinflammatory effects. Consistently, its secretion level in the circulatory system is also elevated in obese models [97]. Recently, resistin was observed to facilitate the formation of foam cells from macrophages by increasing lipid uptake [98], which was induced by the upregulation of scavenger receptors (SR-1 and CD36) [99]. In addition, resistin upregulates the generation of proinflammatory cytokines, including TNF-α and IL-12, in macrophages through an NF-κB-dependent pathway [100]. Therefore, resistin may provide novel insights into the crosstalk among obesity, macrophages, and cancer. Nicotinamide phosphoribosyltransferase (NAMPT, also known as visfatin), a major enzyme of the NAD salvage pathway, was also discovered to mediate the polarization of macrophages [101]. The extracellular form of eNAMPT promotes the production and secretion of IL-6, TNF-α, and CCL2, thereby inducing the M1 polarity of monocytes [102]. In contrast, the ability of eNAMPT to drive M2 polarization in monocytes has also been demonstrated, as treatment with eNAMPT in monocytes upregulated the expression of CD163 and CD206, markers of M2 macrophages [103].

#### Insulin/insulin-like growth factor (IGF)-1

Insulin resistance is common in obese patients and is potentially regulated by macrophage functions in adipose tissue and carcinogenesis [104]. Congruously, insulin, IGF-1 and IGF-2 have been discovered to be elevated in overweight individuals [105]. Insulin is only generated and released by pancreatic β cells, while IGF-1 is mainly generated in the liver [106]. In the case of insulin resistance, peripheral macrophages are polarized toward an M2-like anti-inflammatory phenotype, showing upregulation of M2 markers but downregulation of proinflammatory factors [107]. Similarly, macrophages undergoing knockout of the insulin receptor gene are equipped with anti-inflammatory behavior, which prevents diet-induced obesity [108, 109]. IGF-1 receptor (IGF-1R) is lower in M1-polarized macrophages; however, genetic deletion of IGF-1R suppresses M1 responses but increases M2 responses [110]. In addition, insulin receptor substrate 2, a substrate of insulin receptor and IGF-1R, inhibits alternative polarization of macrophages in vivo [111]. Overall, the suppression of insulin signals in peripheral macrophages facilitates the M2-like phenotype.

### Extracellular vesicles

EVs are a type of vesicle that can be used for cell-to-cell communication to interact with neighboring or distant target cells [112]. Indeed, EVs contain microRNAs, mRNA, proteins, and DNA, among other molecules that can alter the phenotype and function of target cells. EV-specific markers are potential biomarkers. For example, obesity is observed to accelerate the release of EVs, demonstrating that obese patients exhibit higher levels of plasma EVs than normal healthy-weight patients. These EVs are mainly derived from adipocytes [113]. These EVs may potentially be involved in the obesity-induced alteration of macrophages. First, retinol-binding protein 4 is enriched in obesity-associated exosome-like vesicles, which further stimulates the differentiation of peripheral blood monocytes into activated M1 macrophages with increased secretion of IL-6 and TNF-α [114]. In addition, exosomes derived from the tumor-adipocyte microenvironment incorporate some specific microRNAs, including miRNA-126, miRNA-144, and miRNA-155 [61, 115–117], which participate in the polarization of macrophages in different manners. More specifically, the miR-126/miR-126* complex suppresses the mRNA expression of stem cell factor 1α (*SCF-1α*) directly. SCF-1α acts as an upstream regulator of CCL2 in breast cancer cells. Blocking the SCF-1α-CCL2 axis inhibits the recruitment of CCR2^+^ macrophages into tumor tissues and further suppresses the metastasis of tumor cells [118]. Additionally, overexpression of miR-144 inhibits the viability of macrophages and suppresses the generation of TNF-α, IL-6, and IL-8 by downregulating TLR2 and p-p65 [119]. Finally, exosomal miRNA-155 from tumor-adipocyte crosstalk can upregulate the release of CCL2 and CCL5 from adipocytes, thereby recruiting macrophages around adipocytes and repolarizing macrophages toward the M2-like phenotype [61]. Taken together, these findings demonstrate that adipocytes in the fatty microenvironment may endow their EV-derived contents with the transitive capacity to convert the environment of macrophages into a protumor niche.

### Gut microbiota

Recently, some studies have demonstrated that changes in gut microbes have a vital effect on the development of obesity [120]. Compared with normal controls, the intestinal microbiota of obese patients changed considerably, which is particularly related to the associations between bacterial richness, macrophage phenotype, and cancer [121, 122]. Specifically, the gut microbiota may be dominated by potential proinflammatory bacteria, such as *Ruminococcus gnavus* or *Bacteroides*, in the case of obesity [123]. Bacteria-derived LPS is a trigger for the onset of obesity-associated inflammation and infiltration of ATMs. For instance, the elevated infiltration of ATMs as well as obesity is caused in normal diet-fed mice treated with LPS for 1 month [124]. In addition, LPS can activate TLR4 in macrophages to upregulate the secretion of proinflammatory cytokines (including TNF-α, IL-6, and CCL2) [125]. The transmission of LPS to tissue cells may be related to the polarization of macrophages from M2 to M1 in the adipose tissue of high-fat diet-fed mice [126]. Additionally, the products derived from gut microbiota induce low-grade inflammation, which activates tissue-resident macrophages and worsens metabolic diseases, such as diabetes, metabolic syndrome, and cancer [127]. For example, trimethylamine-N-oxide has a positive effect on the reverse cholesterol transport pathway in macrophages and promotes M1 polarization via NOD-like receptor (NLR) family protein (NLRP3) inflammasome activation [128]. Furthermore, G-protein-coupled bile acid receptor 1 (GPBAR-1), the receptor of secondary bile acids, is enabled to modulate energy homeostasis as well as macrophage activation. After being activated by secondary bile acids, GPBAR-1 induces a partial switch from the M1 to M2 subtype for macrophages [129]. Short-chain fatty acids (SCFAs) have also been confirmed to mediate macrophage polarization. In the murine alveolar macrophage MH-S cell line, SCFAs (acetate, butyrate, propionate) decrease the protein or mRNA expression of M2-associated genes in a dose-dependent manner. SCFAs inhibit M2 polarization in MH-S cells, likely by activating G-protein-coupled receptor 43 and inhibiting histone deacetylase [130]. Therefore, the potential links between the microbiome, obesity, and cancer show an emerging and promising direction, and macrophages may be a pivotal mediator.

## Role of ATMs in cancer progression

As the dominant subpopulation of tissue-resident myeloid cells, macrophages not only have a crucial effect on the maintenance of tissue homeostasis but also have a response to adipose tissue and malignant growth [131, 132]. Macrophages are a heterogeneous cluster of immune cells regarding their origin and biology in different tumor types. Generally, the high levels of macrophages surrounding the tumor are presumed to be derived from monocyte precursors and are generally associated with a poor prognosis [133]. However, tissue-resident macrophages are also involved in malignant progression.

Tissue-resident macrophages also exhibit substantial heterogeneity in tissue-specific ways [134]. Recent studies have shown that liver-resident macrophages, Kupffer cells, can endocytose malignant cells to suppress tumor growth [135, 136]. Conversely, macrophages in the brain (microglia) and pancreas accelerate tumor proliferation [26, 137]. Mechanistically, tissue-resident macrophages surround cancer cells early during tumorigenesis to activate epithelial-mesenchymal transition, and they also facilitate the function of T regulatory cells to promote tumor immune escape. However, during tumor growth, macrophages are redistributed at the periphery of the TME and are mainly substituted by monocyte-derived macrophages in mouse and human non-small cell lung carcinoma lesions [27]. Hence, the effect of tissue-resident macrophages and monocyte-derived macrophages on cancer development remains complex. Here, we summarize some studies that investigated the effect of ATMs on malignant tumor progression, angiogenesis, and immune escape (Fig. 1c–e).

### Proliferation and metastasis

ATMs are a mixed type of macrophages mainly composed of tissue-resident macrophages located in adipose tissue and monocyte-derived macrophages, and they are also associated with poor prognosis and aggressive growth [138, 139]. As a site containing a large amount of visceral adipose tissue, the omentum is one of the most common metastatic sites for gastrointestinal and ovarian cancer [140]. In particular, the omentum is early and robustly disseminated by ovarian cancer cells, which represents a poorer prognosis and higher aggressiveness [141, 142]. Omentum-resident macrophages are mainly of embryonic origin and overexpress CD163 and Tim4. Tim4 is regarded as a phosphatidylserine receptor involved in the endocytosis of apoptotic cells [143] and is a special biomarker of tissue-resident macrophages [134, 144]. CD163^+^Tim4^+^ macrophages play a specific role in the invasive progression of metastatic ovarian cancer [139]. The genetic and pharmacological selective depletion of CD163^+^Tim4^+^ macrophages in the omentum prevented the progression and metastasis of ovarian cancer [139]. Furthermore, genetic depletion of the autophagic mediator FAK family-interacting protein reduces the infiltration of Tim4^+^ ATMs and inhibits tumor growth in a T-cell-dependent manner [138].

The TME has fundamental effects on tumor growth and metastasis. The reciprocity between adipocytes and ATMs is regulated under obesity in the TME. Obesity reprograms the metabolic and polarizable phenotype of ATMs. For instance, macrophages remodeled by obesity are predominant ATMs in obese mammary adipose tissue to support tumorigenesis. These macrophages secrete IL-6 to promote stem-like properties via interaction with glycoprotein 130 on triple-negative breast cancer cells [82]. Consistently, weight reduction reverses the roles of obesity on macrophage reprogramming and oncogenesis [82]. On a high-fat diet, ATMs can communicate with white adipocytes via the production of the platelet-derived growth factor (PDGF) ortholog PDGFcc. Indeed, PDGFcc expression is related to poor prognosis in breast cancer [145].

The crosstalk between adipocytes and ATMs in the TME could partly explain the molecular mechanism. Our previous study indicated that CCL2, CCL5, and their receptors linking adipocytes and ATMs promote tumorigenic progression. The elevated expression of CCL2 and CCL5 is mainly secreted by adipocytes, where cancer-derived exosomal miRNA-155 facilitated the production and secretion of CCL2 and CCL5 from adipocytes by modulating the activation of the SOCS6/STAT3 pathway. Consistently, the eliminated macrophages, suppressing the function of STAT3 or chemokines and their receptors, significantly inhibited the tumor proliferation induced by adipocytes [61].

### Angiogenesis

Obesity also plays a role in the angiogenesis induced by macrophages. Obesity results in the activation of the NLR family CARD-containing protein 4 inflammasome in macrophages to increase their infiltration and produce IL-1β [146]. In turn, IL-1β contributes to elevated angiogenesis and tumor progression by upregulating angiopoietin-like 4 from adipocytes in an approach dependent on MAP kinase and NF-κB activation [146]. Adipocytes recruit and excite macrophages through the CCL2/IL-1β/CXCL12 pathway during obesity. In turn, activated macrophages increase angiogenesis to promote cancer progression [147].

### Immune escape

During the process of immune escape, macrophages with high levels of Tim4 are associated with lower numbers of CD8^+^ T cells in hydrothorax and ascites from patients with cancer. It has been demonstrated that phosphatidylserine is mainly upregulated in proliferated and cytotoxic antitumor CD8^+^ T cells, and Tim4^+^ macrophages can integrate with T cells with overexpressed phosphatidylserine to sequester them away from tumor targets and suppress their proliferation [148]. Furthermore, blocking Tim4 enhances the antitumor effectiveness of programmed cell death-1/programmed cell death-ligand 1 (PD-1/PD-L1) blockers and adoptive T-cell therapy in mouse models [148]. Thus, targeting Tim4^+^ ATMs through neutralizing antibodies may potentially provide therapeutic benefits and improve the efficacy of immunotherapies in human cancers.

Regarding the metabolic interplay between adipocytes and ATMs in the TME, adipocytes overexpress fatty acid binding protein 4, which promotes lipid transfer from adipocytes to macrophages and activates IL-6/STAT3 signaling through upregulation of the NF-κB/miR-29b pathway, thereby enhancing tumor proliferation and invasiveness [149]. Furthermore, the long-chain unsaturated fatty acids, specific oleate, amplify the immunosuppressive effects of TAMs. Mechanistically, the enriched lipid droplets and oleate induce polarization of macrophages into CD206^+^ suppressive cells by hyperphosphorylated mTOR complex 2 (mTORC2) at serine 2481 [150].

## Role of ATMs in potential cancer therapy

Given the importance of the dynamic interplay between adipocytes and ATMs in cancer progression, potential therapeutic strategies have attracted considerable interest. The main strategies are divided into those that improve the systemic status and those that disrupt intercellular communications in the TME.

One strategy to improve the systemic status is caloric restriction mimetics (CRMs). CRMs are defined as compounds that simulate the physiological and biochemical changes of caloric restriction to relieve age-associated diseases, including cancer and obesity [151, 152]. Likewise, CRMs, including isobacachalcone, 3,4-dimethoxychalcone, and picropodophyllin, enhance anticancer immunosurveillance and amplify the efficiency of chemoimmunotherapy [153–155]. Caloric restriction benefits metabolism and promotes healthy aging and lifespan in different species [156, 157]. For example, dietary restriction activated p38 signaling and the downstream translation factor ATF-7, which acts as an immune-metabolic pathway that responds to bacterial and nutrient signals, thereby stimulating the conserved innate immunity pathway to extend the lifespan in *Caenorhabditis elegans* [158]. Caloric restriction downregulates the number of lymphocytes and monocytes in blood without affecting responses to vaccines or infections in healthy humans [159]. Similarly, fasting in humans reduced monocytes and dendritic cells in blood but increased the number of proinflammatory Ly6Chi monocytes in the bone marrow [160]. The very-low-carbohydrate diet promotes the production of ketone bodies in healthy humans, which enhances the activity of CD4^+^, CD8^+^, and regulatory T cells, thereby improving human T-cell immunity [161]. Regarding ATMs, the caloric restriction could increase the level of neuropeptide FF in plasma, which promotes the proliferation of ATM and induces M2 activation [162]. Thus, CRMs as a single and/or combinatory approach are potentially applied as an effective strategy against age- or obesity-promoting tumorigenesis.

Other therapeutic targets involved in ATMs and their interaction with other cells have been previously reviewed [22]. Many agents have been explored to regulate ATMs. To target ATM survival, PLX5622, a small-molecule inhibitor of colony-stimulating factor 1 receptor (CSF1R), has been demonstrated to eliminate macrophages regardless of their origin [163]. Combining various CSF1R inhibitors with CD40 agonists effectively depletes macrophages in the TME to inhibit tumor growth [164]. Furthermore, CSF1R inhibition combined with radiation or chemotherapeutic agents markedly improves T-cell responses in animal models [165, 166]. CSF1-CSF1R blockers enhance the therapeutic effectiveness of diverse immunotherapies, such as PD-1/PD-L1 or cytotoxic T lymphocyte antigen 4 antagonists, CD40 agonists, and adoptive T-cell therapy [164, 167, 168]. Given the activation of JAK/STAT signaling and the pathways for angiogenesis in these ATMs [139], inhibition of JAK/STAT signaling or blockade of angiogenesis may be potential therapeutic targets. Finally, epigenetic regulation has an important effect on the proliferation and polarization of ATMs [169]. For example, the transcription factor c-Myc promotes M2 macrophage differentiation by binding to the acetyltransferase p300 [170]. A selective inhibitor of IIa histone deacetylase polarizes macrophages into an antitumor phenotype that sensitizes the tumor to immune checkpoint blockade and chemotherapy in a T-cell-dependent manner [171]. Histone deacetylase 3 deacetylates histone tails in the macrophage genome, suppressing the expression of many IL-4-regulated genes associated with alternative activation of macrophages [172]. Considering the different effects of bone marrow-derived versus tissue-resident macrophages, the deficiency of tissue-resident macrophages may result in some adverse consequences and should be deliberately implemented.

## Conclusions

Plasticity and diversity are well-known characteristics of macrophages. ATMs play a vital role in the progression of obesity-related cancers. Progress has been made in defining the surface phenotype, activating signals, and molecular pathways associated with different forms of ATM activation. Although many studies have demonstrated that ATMs profoundly reprogram their functions in obesity-related cancers, ATM-targeted treatments according to different activation mechanisms have not been thoroughly investigated.

Except for scRNA-seq, some new experimental approaches have been applied in the study of microenvironments, including microfluidics, 3D spheroids, and organs-on-chips. Based on droplet microfluidics, spheroids are filled with a novel hydrogel to promote cell adhesion and aggregation, and this system is composed of cancer cells, fibroblasts, and lymphocytes for dynamic analysis of cellular interactions, proliferation, and therapeutic efficacy and has been used in lymphoma research [173]. Compared with the common Transwell coculture system, 3D spheroids model the interactions among multiple cell types in the TME in a better manner [174]. Microfluidic cell culture technology promoted the production of human organs-on-chips, which has been applied to model cancer cell behavior in organ microenvironments in vitro. This approach facilitates the analysis of the effects on tumorigenesis, tumor progression, and responses to therapy [175].

This review discusses four potential mechanisms for ATM polarization in obesity-related cancers: (1) Obesity-induced metabolic alterations regulate the recruitment, differentiation, and polarization of macrophages in the TME. (2) Secreted molecules in adipose tissues drive tumor progression through alternative pathways of ATM infiltration and polarization. (3) Following the transfer of EV cargoes derived from adipose tissues, ATMs undergo polarization shifts in both phenotype and function, thus being more effective in promoting tumor growth. (4) The secretion of inflammatory cytokines, which stimulate the recruitment and polarization of ATMs, is also mediated by the gut microbiota and its metabolites. However, only a few metabolites involved in the process of driving the polarization of ATMs have been studied. Other tumor-secreted or obesity-induced metabolites have not yet been fully investigated.

Furthermore, many questions remain regarding the mechanisms by which cytokines, adipokines, and hormones regulate macrophage polarization. Because of the complex interaction between cytokines and metabolites, there is still much to be explained regarding the physiological response of macrophages in obesity-mediated TME. Further elucidation of the impact of EVs on macrophages in the adipose TME seems to be a promising field for further investigation to address a variety of obesity-associated tumors. The exact effect of gut microbial metabolites on the pathogenesis of obesity-related cancers also remains to be investigated. Hence, a better understanding of ATM diversity and activation will provide new strategies for the treatment and prevention of obesity-related cancers.

## Data Availability

Not applicable.

## Abbreviations

ATMs: Adipose tissue macrophages
BCAAs: Branched-chain amino acids
BCAT: Branched-chain aminotransferase
BCKA: Branched-chain alpha-keto acid
CCL2: Chemokine (C-C motif) ligand 2
CCL5: Chemokine (C-C motif) ligand 5
CCR2: C-C chemokine receptor 2
CD36: Cluster of differentiation 36
CLSs: Crown-like structures
CRMs: Caloric restriction mimetics
CSF1R: Colony-stimulating factor 1 receptor
EVs: Extracellular vesicles
GPBAR-1: G-protein-coupled bile acid receptor 1
Gpr132: G-protein-coupled receptor 132
HIF-1α: Hypoxia-inducible factor 1α
HSC: Hematopoietic stem cell
IFN-γ: Interferon gamma
IGF: Insulin-like growth factors
IGF-1R: IGF-1 receptor
IRG1: Immune responsive gene 1
LAMs: Lipid-associated macrophages
LPS: Lipopolysaccharide
Ly6C: Lymphocyte antigen 6C
MCs: Mast cells
mTOR: Mammalian target of rapamycin
mTORC2: mTOR complex 2
NAMPT: Nicotinamide phosphoribosyltransferase
NF-κB: Nuclear factor kappa B
NLRP3: NOD-like receptor (NLR) family protein
PD1: Programmed cell death-1
PDGF: Platelet-derived growth factor
PD-L1: Programmed cell death-Ligand 1
PPARγ: Peroxisome proliferator-activated receptor-gamma
SCF-1α: Stem cell factor 1α
SCFA: Short-chain fatty acids
scRNA-seq: Single-cell RNA sequencing
TAM: Tumor-associated macrophage
TAME: Tumor-adipose microenvironment
TCA cycle: Tricarboxylic acid cycle
TLR4: Toll-like receptor 4
TME: Tumor microenvironment
TNF-α: Tumor necrosis factor-α
TNFR: TNF-α receptor
